# Personality pathology in adolescence: relationship quality with parents and peers as predictors of the level of personality functioning

**DOI:** 10.1186/s40479-022-00202-z

**Published:** 2022-10-19

**Authors:** Gabriele Skabeikyte-Norkiene, Carla Sharp, Paulina Anna Kulesz, Rasa Barkauskiene

**Affiliations:** 1 Institute of Psychology, Faculty of Philosophy, Vilnius University, Universiteto st. 9, 01513 Vilnius, Lithuania; 2 Department of Psychology, University of Houston, Houston, USA; 3 Texas Institute for Measurement, Evaluation, and Statistics (TIMES), University of Houston, Houston, USA

**Keywords:** Level of personality functioning, Alternative Model for Personality Disorders (AMPD), ICD-11, Adolescence, Relationship quality, Network of relationships

## Abstract

**Background:**

The dimensional approach to personality pathology opens up the possibility to investigate adolescence as a significant period for the development of personality pathology. Recent evidence suggests that symptoms of personality pathology may change during adolescence, but the negative consequences such as impaired social functioning persist later on in life. Thus, we think that problems in social functioning may further predict personality impairments. The current study aimed at investigating the role of relationship quality with parents and peers for the prediction of the level of personality functioning across adolescence. We hypothesized that 1) relationship quality with both parents and peers will significantly account for the level of personality functioning in adolescence and 2) the importance of relationship quality with peers for the relation to impairments in personality functioning will increase with age.

**Methods:**

A community sample consisting of 855 adolescents aged 11–18 (M = 14.44, SD = 1.60; 62.5% female) from different regions in Lithuania participated in this study. Self-report questionnaires included the *Levels of Personality Functioning Questionnaire* to investigate personality impairments and the *Network of Relationships Questionnaire* to assess the quality of dyadic relationships.

**Results:**

Discord in the parent, but not peer relationships, was related to a more severe level of personality functioning across adolescence. Lower levels of closeness with parents accounted for higher impairments in personality functioning. The importance of closeness with peers for the explanation of the level of personality functioning increased with age.

**Conclusions:**

During the sensitive period for the development of a personality disorder, relationship quality with the closest adults and peers both remain important for the explanation of impairments in personality functioning.

**Supplementary Information:**

The online version contains supplementary material available at 10.1186/s40479-022-00202-z.

## Background

The last decade was marked by changes in the conceptualization of personality pathology, which was accelerated by the criticism of the existing categorical model of personality disorders (PD). A categorical model is a symptom-based approach, which implies that personality pathology is distinct from normative personality and this allows the categorization of distinct syndromes [1]. However, long debates on the validity of widely used categories resulted in a proposal of a new approach [2, 3], namely the Alternative DSM-5 model for personality disorders (AMPD). In contrast to the categorical approach, the AMPD requires evaluation of a unidimensional severity criterion represented by maladaptive self and interpersonal functioning as the entry criterion (Criterion A; Level of Personality Functioning) for the diagnosis of personality disorder [1]. Similarly, the dimensional approach to personality disorder proposed in ICD-11 posits the severity of personality dysfunction as the entry criterion for the evaluation of personality disorder [4]. The construct of personality functioning and the severity continuum in AMPD and ICD-11 are both defined through impaired identity function and self-directedness as well as one’s capacity for empathy and intimacy [1]. Thus, both diagnostic systems include similar features and allow one to identify the personality disorder through the evaluation of impairments in individual functioning, which range from healthy to severely impaired. Psychological capacities for self and interpersonal functioning develop over the lifespan [5], and at this point, both diagnostic classifications provide an option for the diagnosis of a personality disorder for adolescents. This opens up the possibility for empirical studies of personality (dys)function in adolescence, which is now considered as a period in which personality disorder usually has its onset and can be validly assessed [6]. Emerging data suggest that assessment of PD through the evaluation of personality functioning is a more developmentally sensitive approach than using a categorical symptom-based approach and may contribute to the early detection of the disorder in adolescence, when the PD may not be fully developed [5]. In that way, self and interpersonal functioning as the main criterion for a personality disorder is seen as emerging and developing in adolescence [7].

The development of the sense of self or identity formation is one of the main developmental tasks throughout adolescence [7, 8], and current knowledge suggests that adolescent relationships have an impact on identity development [9] in a way that the development of self builds on a strong foundation of prior and continuing attachment security with parents and high-quality relationships with peers [7, 10]. Adolescence also stands out as a period with developmental cascades in social cognition and competence which includes not only self and other perception, but also the perception of the interpersonal processes that become more mature and capture the extended social network of close friendships and romantic relationships [11]. Formulating one’s worldview and creating identity is affected by the young person’s relationships with family, friends, peers, and teachers, and the ability to maintain and self-disclose in a relationship is essential to forming a coherent sense of self or identity formation [12–14]. In this developmental period, there is a normative shift towards peers for intimacy and attachment, and peer relationships become more important. Striving for autonomy is an important task in adolescent identity development, often marked by increased conflicts with parents [15, 16]. Thus, past and present relationships with family and peers appear as important factors for the development of self-function in adolescence.

Existing data indicate the importance of interpersonal processes on the development of the capacity for interpersonal functioning. First, attachment as well as relationship quality with parents and friends are found to be important for the development of the capacity for empathy [17, 18]. Second, the maintenance of relationships through self-disclosure in a relationship helps to build the capacity for reciprocity [13, 19], while attachment security predicts higher levels of intimacy and general social competence in adolescence [20, 21]. On the other hand, conflictual and dominant relationships may impair the development of intimacy [20]. Thus, adolescent relationships play a prominent role as the source of support and provide the context for social learning experience [11], while poor social functioning may pose a risk for a more impaired level of interpersonal functioning.

Evidence from different studies suggests that poor social functioning in both parental and peer relationships, peer rejection, or victimization creates a powerful threat to mental health [22–25]. Poor relationships with parents, including coercive parenting, parent–child discord [26, 27], diminished attachment [28], impaired boundaries [29], and negative interactions with peers and mothers [30] are associated with the development of a borderline personality disorder (BPD) in adolescence. Data suggest that being exposed to relational, psychological, or sexual violence is associated with increases in borderline personality disorder symptoms throughout adolescence [31–33]. Researchers indicate that personality disorders are associated with poorer social and occupational functioning [34, 35], and while the symptoms of a personality disorder may wax and wane through adolescence, problems in social functioning are relatively stable and have long-term consequences [30]. Given that personality disorders are interpersonal in origin, it is reasonable to hypothesize that social problems may not only be seen as the consequence of a disorder but also as a risk factor for further impairments in the development of personality.

Vanwoerden, Franssens, Sharp & De Clercq (2021) recently provided evidence that pre-adolescent social problems rated by parents predict lower levels of personality functioning (self-function) in early adulthood, which provides support for the idea that problems in social functioning have repercussions not only for other relationships, but may also have an impact on the development of personality dysfunction [36]. However, the study provides personality functioning scores in early adulthood, with social functioning scores attained at age 12. Therefore, little is known about whether social functioning also associates with personality functioning in adolescence itself.

Additional limitations of previous work include that the vast majority of the conducted studies cover categorical concepts of personality disorders, mostly borderline personality disorder. Having in mind the recent switch from the categorical to dimensional approach towards personality pathology, research investigating the factors related to the level of personality functioning is necessary, and has, as yet, not been undertaken. Second, existing research on social functioning mostly includes only one type of relationship (mothers/siblings/peers, etc.), which does not capture the complexity of the adolescent’s social world [37]. Currently, significant effort has been put toward the analysis of the parent–child relationship‘s role in the child’s personality development [38], but the way in which peer relationships in adolescence interact with the maturation of personality is still unclear [11, 39]. Third, previous studies have not taken into account age cohort effects on outcomes.

Highlighting these limitations, the aim of this study was to explore the role of subjective positive and negative relationship quality with parents and peers for the prediction of the level of personality (dys)function in adolescence. Since personality disorders have high comorbidity rates with other psychopathology, including internalizing and externalizing difficulties [40, 41], we have decided to include general psychopathology as well, which will allow us to understand the association between relationship quality and personality functioning, independent from other forms of psychopathology. We expect that even though adolescence is marked by a shift from reliance on parents towards reliance on peers, increased levels of conflicts with parents are common in adolescence [15, 16], and the negative interactions with parents will remain significant in explaining a more severe level of personality functioning throughout adolescence. Second, since parents and peers might provide different and unique contexts for identity development [38], we hypothesize that lower levels of closeness with peers and parents will both emerge as important factors that account for more impaired personality functioning. Finally, time spent with peers and intimacy between peers increases during adolescence [17], so we expect that the role of the relationship quality with peers in relation to the level of personality functioning will become more important as adolescents grow older.

## Methods

### Participants and procedures

The sample consisted of 855 adolescents aged 11–18 (M = 14.44, SD = 1.60; 62.5% female and 37.5% male) who were enrolled through public schools covering several cities (37.2%), towns (40.9%) and rural areas (21.9%) in Lithuania. More than half of the participants (66.5%) reported that their parents were married. Participants also reported that their parents were divorced (18.5%) or that the status of the family relationship was “other” (10.90%).

We used the non-probability quota sampling method to form a sample of evenly distributed different age groups and areas in Lithuania. Invitations to participate in the study and written parent consent forms were distributed to pupils through the selected schools. Only adolescents whose parents gave written consent participated in the study. All participants were informed about their right to withdraw from the study at any time.

The study was conducted by trained research assistants during school hours in small groups of pupils who were asked to fill out the questionnaires. Part of the study was conducted during the lockdown due to Covid-19. According to the World Health Organization, increased levels of psychological problems might be seen during and after the pandemic, which might also have an impact on the participants‘ responses. The presented cross-sectional data is part of the large longitudinal study in Lithuania, that addresses different aspects of adolescent personality and psychosocial functioning. The full study protocol was approved by the Psychological Research Ethics Committee at Vilnius University.

### Measures

#### Personality pathology

The level of personality functioning was assessed with the culturally adapted Lithuanian version of the DSM-5 based instrument *Levels of Personality Functioning Questionnaire* (LoPF-Q 12–18) [42]. It is a 97-item self-report instrument with a 5-step response format (0 = no to 4 = yes) with higher scores indicating a more severe level of impairment in personality functioning and a higher risk for a current personality disorder. The questionnaire allows assessing dimensionally the total score of personality dysfunction as well as adaptive function or disturbances in the self and interpersonal domains. The original questionnaire was developed by a research group in Basel University clinics, Switzerland. The adaptation procedure for the Lithuanian version of the LoPF-Q 12–18 [43] included the translation and back-translation of the items based on the discussion with the instrument authors. Subsequently, the pilot and main empirical studies were conducted to ensure the necessary psychometric qualities of the questionnaire. Adolescents (*N* = 362; 83% school-based sample; 17% clinical sample) participated in the main empirical study. The LoPF-Q 12–18 scores differentiate adolescents with 5 or more BPD symptoms from the school-based sample of adolescents with BPD (Cohen’s d = 1.2), which proved the clinical validity of the LoPF-Q 12–18 (unpublished dataset). Based on the previous discussions about the LoPF-Q structure and existing attempts to identify the most valid structure of the instrument, we have decided to use a total LoPF-Q score as a unidimensional measure of personality functioning [44, 45]. In the current study, the internal consistency was excellent for the total scale (α = 0.97).

#### Relationship quality

*Network of Relationships Questionnaire-Relationship Qualities Version* (NRI-RQV) [37] was used to assess the subjective quality of adolescent relationships. It is a self-report instrument with 30 items and a 5-step response format (1 = never or hardly at all to 5 = always or extremely much). Items are then divided into subscales in which a higher mean on a subscale level indicates a higher expression of the specific quality. The chosen version of the questionnaire employs a set of relationship qualities that describes the supportive and discordant qualities of relationships with parents and peers. The features assessed are more of a behavioral or observable nature and are rated on the scale “how often” do you experience particular features rather than reveal attitudes and insights. In our study adolescents evaluated their current relationships with their best friend and both parents separately. Parental scales were then transformed into one scale by extracting the mean of the relationship quality with both mother and father. In this study, only the two broad scales of positive (closeness) and negative (discord) qualities of the relationships were evaluated to capture the different valence of adolescents’ interactions. The positive qualities scale was constructed of several aspects of relationships, including companionship, disclosure, satisfaction, emotional support, and approval. Similarly, negative qualities were defined through subjective pressure, conflict, criticism, dominance, and exclusion in the specific relationship. The original version of the measure showed good internal consistency with Cronbach α ranging from 0.89 to 0.93 for the closeness scale and 0.80-0.84 for the discord scale [46]. The questionnaire was translated into Lithuanian language by two independent experts at the Developmental Psychopathology Research Center at Vilnius university, and after a thorough discussion, the final version of the questionnaire was prepared for the study. The internal consistency was high both in closeness (α = 0.89) and discord (α = 0.87) in peer relationships as well as parent relationships (accordingly, α = 0.92 and α = 0.91).

#### General psychopathology

*Youth Self-Report* (YSR 11/18) [47] was used to measure internalizing and externalizing difficulties which will be further reported as general youth psychopathology. It is a 112-item self-report instrument with a 3-point answer scale (0 = not true, 1 = somewhat or sometimes true, 3 = very true or often true). The instrument is fully standardized for use in a Lithuanian sample [48]. Internal consistency of the used subscales in this study is high, with Cronbach α being equal to 0.94 for internalizing and 0.89 for externalizing difficulties.

### Statistical analyses

Before addressing questions of interest, we computed descriptive statistics (means and standard deviations), and Pearson correlations to examine bivariate relations between variables used in subsequent analyses. The False Discovery Rate (Benjamini & Hochberg, 1995) at the level of 0.05 was used as a correction for multiple computed correlations. Multiple regression models with fixed predictors were computed to examine the effects of subjective positive and negative relationship qualities with parents and peers on the level of personality (dys)function in adolescence. The level of personality (dys)function was a continuous outcome. On the predictor side of the models, continuously distributed negative and positive relationship qualities, as well as internalizing and externalizing difficulties were grand mean centered at a mean value for 15-year-old participants. Gender, internalizing difficulties, and externalizing difficulties were treated as covariates in computed models. Age and relationship quality interaction with age were included to examine whether the level of personality (dys)function changes over time and whether relations between personality (dys)function and relationship quality are moderated by age, respectively. A statistically significant interaction of age and positive relationship qualities with peers was depicted using a line plot. In the plot, continuously distributed age and positive relationship qualities with peers were categorized into two or three categories (respectively) to simplify plotting. Specifically, we computed low (below 1 standard deviation), average (mean value), and high (above 1 standard deviation) values for positive relationship qualities with peers using mean and standard deviations. A similar process was repeated for the age variable. After obtaining the aforementioned values, we used these constants in regression equations to obtain respective intercepts that were subsequently connected using lines on the plot. Regression models were separately computed for negative and positive relationship qualities. Regression diagnostics were examined to ensure that regression assumptions are met. All analyses were conducted using the *Statistical Package for Social Sciences* (SPSS) v. 23.

## Results

### Preliminary findings

The current sample covers a broad age span which ranges from 11 to 18 years old. Descriptive statistics of used measures across age span (grouped into 6 age groups) are presented in Table 1. The mean LoPF-Q total score was the highest in middle adolescents group (M = 150.40 at age 15) and slightly lower for younger (M = 141.36 at age 11–12, M = 148.45 at age 13 and M = 146.64 at age 14) and older adolescents (M = 147.10 at age 16 and M = 146.63 at age 17–18). Levels of closeness with parents were found to be the highest for early adolescents (M = 3.73) and lowest for older adolescents (M = 3.38). Levels of discord in relationship with parents were found to be the highest from early to middle adolescence (M = 2.05 at age 11–12 and M = 2.19 at age 14) and lower in late adolescence (M = 2.02 at age 17–18). The mean score of closeness in relationship with peers differed across each adolescents group (M = 3.64 at age 11–12; M = 3.83 at age 13; M = 3.82 at age 14; M = 3.68 at age 15; M = 3.91 at age 16; M = 3.69 at age 17–18). Discord in peer relationships was the highest among early and middle adolescents (M = 1.79 at age 11–12 and M = 1.89 at age 15) and lower for late adolescents (M = 1.57 at age 17–18). Scores of internalizing difficulties were the lowest in the youngest adolescent group (M = 15.12 at age 11–12) and highest for the oldest adolescents (M = 20.88 at age 17–18). Mean levels of externalizing difficulties were the lowest for early adolescents and late adolescents (M = 9.76 at age 11–12), and the highest for middle adolescents (M = 14.23 at age 16 and M = 13.69 at age 17–18).

**Table 1:** Descriptive statistics by age groups

Measure	LoPF_Q total score	NRI parent closeness	NRI parent discord	NRI peer closeness	NRI peer discord	Internalizing difficulties	Externalizing difficulties
Age group	M (SD)	M (SD)	M (SD)	M (SD)	M (SD)	M (SD)	M (SD)
11–12 (*n* = 128)	141.36 (56.50)	3.73 (.88)	2.05 (.86)	3.64 (.90)	1.79 (.69)	15.12 (12.31)	9.76 (7.95)
13 (*n* = 141)	148.45 (58.28)	3.61 (.88)	2.11 (.75)	3.83 (.80)	1.82 (.75)	19.11 (12.27)	10.79 (7.72)
14 (*n* = 153)	146.64 (59.03)	3.39 (.98)	2.19 (.88)	3.82 (.91)	1.86 (.72)	18.76 (13.11)	11.52 (8.16)
15 (*n* = 166)	150.40 (58.56)	3.28 (1.01)	2.11 (.75)	3.68 (1.20)	1.89 (.74)	19.88 (12.93)	14.14 (8.95)
16 (*n* = 186)	147.10 (59.14)	3.14 (1.01)	2.11 (.75)	3.91 (.95)	1.79 (.66)	20.49 (12.24)	14.23 (8.57)
17–18 (*n* = 81)	146.63 (58.74)	3.38 (.99)	2.02 (.81)	3.69 (1.08)	1.57 (.69)	20.88 (13.12)	13.69 (9.84)

Although mean values for outcomes, predictors, and covariates are reported in the table by age, regression analyses included age as a continuous measure rather than categorical. We combined ages 17 and 18 into one group because there was only 1 participant who was 18 years old. Similarly, we combined ages 11 and 12 into one group because there were 11 participants who were 11 years old

*LoPF-Q* Levels of personality functioning questionnaire, *NRI* Network of relationships inventory, *closeness* Positive relationship qualities, *discord* Negative relationship qualities

Relations between personality (dys)function, relationship quality, internalizing and externalizing difficulties, and age are presented in Table 2. Older age was related to lower levels of closeness with parents (*r* = -0.22; *p* < 0.004) and internalizing (*r* = 0.12; *p* < 0.002) and externalizing (*r* = 0.19; *p* < 0.007) difficulties. Domains of positive (*r* = -0.51, *p* < 0.002) and negative (*r* = 0.42, *p* < 0.001) relationship quality with parents were significantly and moderately correlated with the total score of personality functioning. Correlations between the positive (*r* = -0.14, *p* < 0.002) and negative (*r* = 0.19, *p* < 0.002) relationship quality with peers and the total score of personality functioning were of small magnitude, but statistically significant, demonstrating that lower levels of closeness and higher levels of discord in parent and peer relationships were related to a more impaired level of personality functioning. Positive relationship quality was correlated with negative relationship quality in peer relationships (*r* = 0.21, *p* < 0.005), which means that a higher level of closeness in a relationship with a best friend was also associated with a higher level of discord. Negative relationship quality was associated with both higher levels of closeness (*r* = 0.09, *p* < 0.01) and higher levels of discord (*r* = 0.46, *p* < 0.004) in relationship with parents. Last, there was a negative association of small magnitude between positive and negative qualities in parent relationships (*r* = 0.10, *p* < 0.01) indicating that higher levels of support are associated with lower levels of discord.

**Table 2:** Pearson correlation coefficients after adjusting for multiple computed correlations using the Benjamini–Hochberg procedure with a false discovery rate of .05

Variable	1	2	3	4	5	6	7	8
1. Age	1	.01	.03	-.07	-.22^a^	-.01	.12^a^	.19^a^
2. LoPF-Q		1	-.14^a^	.19^a^	-.51^a^	.42^a^	.72^a^	.48^a^
3. NRI peer closeness			1	.21^a^	.29^a^	-.01	-.01	.01
4. NRI peer discord				1	.09^a^	.46^a^	.12^a^	.16^a^
5. NRI parent closeness					1	-.10^a^	-.49^a^	-.39^a^
6. NRI parent discord						1	.31^a^	.34^a^
7. Internalizing difficulties							1	.60^a^
8. Externalizing difficulties								1

^a^Statistically significant correlations after adjusting for multiple computed correlations

### The effects of relationship quality on the level of personality (dys)function

Table 3 presents regression coefficients and model fit indices of the computed models. The model focusing on negative aspects of parent and peer relationships, controlling for internalizing and externalizing difficulties, and gender accounted for 58% of the variance in personality (dys)function (LoPF-Q). Findings suggested that only negative relationship quality in interactions with parents (β = 0.191, *p* < 0.001) was related to higher impairments in adolescents’ personality functioning, when controlling for gender, internalizing, and externalizing difficulties. Age (β = -0.070, *p* = 0.005) and internalizing (β = 0.64, *p* < 0.001) difficulties were related to the LoPF-Q scores, such that older age and higher levels of internalizing problems accounted for higher impairments in personality functioning. Moreover, negative relationship quality with peers or interactions with age were non-significant in explaining LoPF-Q scores.

**Table 3:** Linear regression models with fixed predictors for the explanation of LoPF-Q total score

	B	*β*	*SE*	t	R^2^	F
**Model 1: negative qualities**					.58	125.84^c^
Age	-2.54	-.07	.89	-2.84^b^		
Gender	-1.79	-.02	3.26	-.55		
Internalizing difficulties	2.95	.64	.15	19.49^c^		
Externalizing difficulties	.27	.04	.21	1.27		
NRI parent discord	14.09	.19	2.28	6.17^c^		
NRI peer discord	3.48	.04	2.48	1.40		
Age x NRI parent discord	-.06	-.001	1.33	-.05		
Age x NRI peer discord	.35	.01	1.52	.23		
**Model 2: positive qualities**					.58	127.52^c^
Age	-4.04	-.11	.91	-4.44^c^		
Gender	-2.12	-.02	3.33	-.64		
Internalizing difficulties	2.77	.60	.16	17.58^c^		
Externalizing difficulties	.44	.06	.21	2.09		
NRI parent closeness	-11.91	-.20	1.79	-6.65^c^		
NRI peer closeness	-3.29	-.05	1.64	-2.01^a^		
Age x NRI parent closeness	-.40	-.01	.95	-.42		
Age x NRI peer closeness	1.93	.05	.96	2.02^a^		

^a^ significant at the level less than .05

^b^ significant at the level less than .01

^c^ significant at the level less than .001

**Fig. 1:**
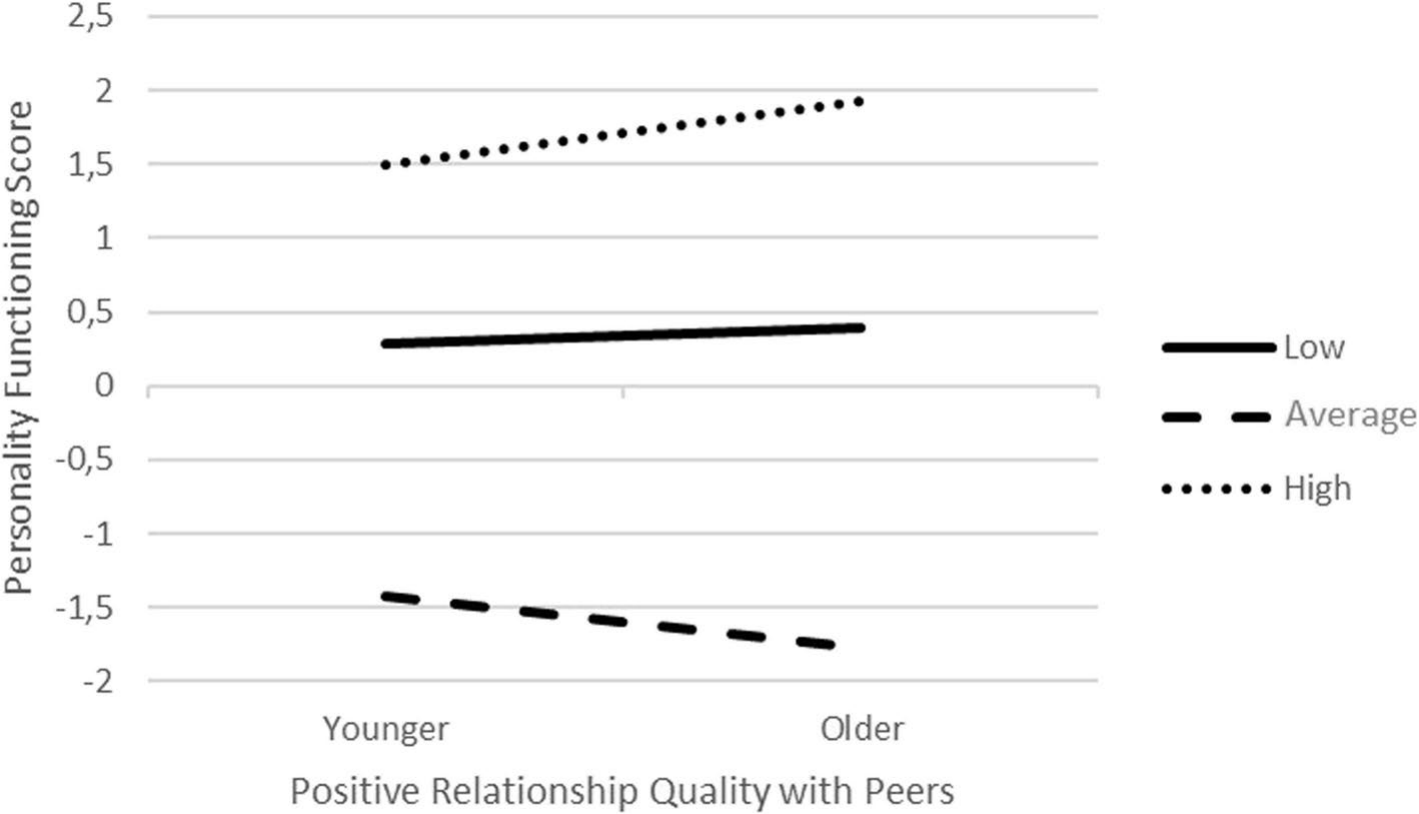
A line plot demonstrating the personality functioning score for low (solid line), average (dashed line), and high (dotted line) positive relationship qualities with peers for older and younger participants. Younger = younger participants; Older = older participants

The model examining the role of positive relationship quality with parents and peers, controlling for internalizing and externalizing difficulties, and gender, accounted for 58% of the variance in personality (dys)function. Positive relationship quality with parents was related to LoPF-Q scores (β = -0.198, *p* < 0.001), controlling for other terms in the model. This finding implies that closeness with parents (regardless of an adolescent’s age) is important in explaining the level of personality functioning. Closeness with parents can be regarded as a stable construct that impacts adolescents' personality functioning across the developmental span. The interaction between age and closeness in peer relationships was also statistically significant (β = 0.052, *p* = 0.04), over and above other terms in the model. As depicted in Fig. 1, the interaction effects were dominated by the main effects such that both younger and older participants with lower (defined as one standard deviation below the average) or higher (defined as one standard deviation above the average) positive relationship quality had higher LoPF-Q scores relative to younger or older participants with average positive relationship quality. Yet, the difference in relations between lower or higher positive relationship quality and LoPF-Q scores, versus relations between average positive relationship quality and LoPF-Q scores was more pronounced for older participants revealing an ordinal type of interaction. Together, these findings suggested that both parental and peer positive relationships are important for the level of personality functioning such that closeness with parents may be seen as a stable quality regardless of the adolescent’s age, while closeness in peer relationships changes as a function of age. Finally, internalizing (β = 0.602, *p* < 0.001) and externalizing difficulties (β = 0.064, *p* < 0.001) also statistically significantly accounted for the LoPF-Q scores, indicating that higher levels of difficulties were associated with higher LoPF-Q scores.

## Discussion

The current study aimed at exploring the role of relationship quality with parents and peers for the prediction of impairment in the level of personality functioning in adolescents. The analyzed data came from a large adolescent community sample covering different areas in Lithuania. This is one of the first studies to examine the dimensional concept of personality pathology in relation to adolescents‘ current subjective social functioning across a broad adolescence age span.

In this study, we conceptualized personality pathology through a dimensional model of personality disorders, which was proposed in DSM-5 and further adapted for use in ICD-11. Emerging data suggest that diagnostic information obtained using DSM-5 assessment tools can be used for making an ICD-11 dimensional personality disorder diagnosis [49], which makes the assessment of the level of personality functioning as a proxy indicator of severity. Thus, the unidimensional concept of severity in personality pathology was assessed using the Levels of Personality Functioning Questionnaire for adolescents (LoPF-Q 12–18) [42], which allows the attainment of a total score of severity in the level of personality functioning. The quality of relationships was seen as a subjective evaluation of behavioral aspects of relationships with a mother, father, and best friend using the Network of Relationships Questionnaire (NRI) [37]. The obtained scores were compiled into positive and negative relationships with parents and peers, reflecting the experienced closeness and discord in these close relationships.

Several findings are notable. First, there were no significant associations between the level of personality functioning and age, indicating that in a community sample, personality functioning was found as a relatively stable construct throughout adolescence. Previous evidence on maladaptive personality traits suggest that features of personality pathology emerge in early adolescence, reach their peak in middle adolescence, and then decrease as adolescents enter adulthood [50]. Similarly, recent research revealed the normative increase in maladaptive identity throughout adolescence, which was closely related to increases in borderline personality features, especially for older adolescents [51]. However, our data catches the wider scope of general severity in personality functioning rather than discrete personality features so it is possible that even though personality features might change, the general level of personality functioning follows a more complex pattern of change.

Next, as expected, we found that negative interactions with parents were related to the more severe level of personality functioning, independently from adolescents' age. This is comparable to previous research showing an association between negative experiences in relationships with parents such as parental control or coercive parenting and aspects of personality pathology [14, 26, 30]. Thus, discordant qualities of relationship with parents stand out as a potentially important factor for the prediction of higher levels of impairment in personality functioning. Of course, given the cross-sectional nature of our data, the direction of influence is not causal and directionality can only be determined by prospective follow-up studies.

Unexpected results were also found – specifically, that discord in peer relationships was not related to the level of personality functioning. Previous studies have provided much evidence supporting the opposite and showing that negative experiences with peers are very important for the development and course of a personality disorder [24, 31, 52, 53]. One of our explanations would be that the negative interactions that we were investigating were not at that extreme level, as victimization (studied in previous samples) would be. Negative interactions with peers that include conflict or criticism in relationships might be more closely related to the normative aspect of discord in relationships, but not direct victimization.

In addition, lower levels of closeness in parent relationships were found to account for higher levels of impairments in personality functioning. Data on categorical personality disorders have shown similar results demonstrating that low maternal emotional support was associated with higher severity of BPD symptoms [54]. It was found that BPD symptoms and parenting practices that are low in warmth might even maintain each other during adolescence [55]. On the other hand, higher maternal support was associated with lower subsequent BPD scores and was seen as a strong protective factor [36]. Higher quality of relationships with father and mother was in general associated with higher adolescent well-being and it seems that interpersonal support can offer some survival strategies that help to build relational capacities in the complicated process of personality maturation [56, 57]. Our findings reveal that lower levels of closeness with parents account for higher impairments in personality functioning, but, however, data suggest that sufficient levels of closeness can also be associated with higher adaptive level of personality functioning.

The most noteworthy finding that emerged in this study was that even though closeness with parents remained important independently from the adolescent’s age, the importance of closeness with peers in explaining the variance in the level of personality functioning increased with age. This is supported by theory on adolescents' social development during childhood since one of the developmental milestones in the transition from parental reliance to autonomy in adolescence is learning to create trustworthy and reliable relationships with peers, which become more important with age [20, 58]. The increasing relevance of peers is important against the background of evidence suggesting that support from family and friends may decrease the risk for internalizing psychopathology, buffer the effects of earlier adverse and bullying experiences, and may even provide context for protection against victimization in the long-term [59, 60].

Another interesting finding was that very low or very high levels of closeness in peer relationships were associated with higher impairments in personality functioning when compared to average levels of closeness with peers. This reveals that not only the lack of closeness might contribute to the development of a personality disorder, but also the elevated levels of closeness which are deviant from the average levels that adolescents usually report. Lazarus (2019) provided similar evidence suggesting that higher levels of support in adolescent romantic relationships predict steeper increases in BPD symptoms across adolescence [61]. These findings report the potential negative influence of overreliance and early involvement in close romantic relationships and our data suggest that overly close relationships with a best friend might also be significant for the development of impairments in personality functioning. On the other hand, it is reported that adolescents who have personality disorders strive for intimacy in relationships and their view toward significant people and relations to them might be distorted or overly idealized [1].

Also, higher levels of closeness in peer interactions were related to higher levels of discord in those relationships. Similar results were obtained in a recent study by Hessels (2022) in which they investigated a clinical sample of adolescents. Authors explain that adolescents at risk for a personality disorder might experience the interactions with a best friend at a more extreme level with friendships providing a ground for both supportive and negative interactions. Since personality disorders are marked by serious disturbances in interpersonal functioning, this was considered as a marker of BPD in the studied sample [30]. However, we investigated a community-based sample so we hypothesize that intense involvement in peer relationships might also be the marker of the normative shift from parent to peer influence that is common for this developmental stage [20, 58]. Also, in another study, the frequency of close contact was found to be associated with the level of conflict in relationships [62] so it is possible that a relationship with a best friend in adolescence is more intense and frequent, which might also lead to both closeness and discord.

To sum up, even though adolescents go through the change of developmental tasks with higher importance being placed on peer relationships, it seems that in the process of the development of personality pathology, not only peer relationships are significant, but relations to parents remain important throughout adolescence. Supporting our findings, McLean and Jennings (2012) state that parents and friends provide unique contexts with different implications in the process of identity development, and while parental relationships are indeed crucial for the construction of internal models of extended relationships, high-quality peer relationships are essential in a way that they may provide a safe place for identity explorations away from parents [18, 38]. We conclude that in our study parent and peer relationships both remain significant and depending on the valence of the relationship, create an important context for the development of a level of personality functioning.

The study has several limitations. First, self-report was used to evaluate the main constructs of the study which capture only the subjective experience of Lithuanian adolescents. Data from several sources of information (e.g. parents, friends) or obtained through qualitative methods would provide additional important information. Second, the conducted study is cross-sectional, which did not allow us to capture the interaction among constructs in time. While our study has developmental implications by comparing different age groups, future studies should include within-person longitudinal samples in order to better explain the possible mechanisms in which adolescent social experiences interact with the level of personality functioning. Third, the current study was launched during the quarantine and lockdown due to the Covid-19 pandemic, which might have an impact on our data, especially regarding evaluations of relationship quality.

## Conclusions

In accordance with the recommendations proposed by Chanen (2017), research is moving towards the identification of the factors that may account for the development of a personality disorder [63]. In the context of a recently developed dimensional model of personality disorders, our data add up to the knowledge about the possible risk and protective factors for the level of personality functioning. Even though we see the shift towards peers for interpersonal support in adolescence and important positive relationships seem promising for a healthier level of personality functioning, discord in parent relationships appears as a stable and significant factor that accounts for higher levels of severity in the level of personality functioning throughout adolescence. Previous data have shown that impaired social functioning is one of the long-term consequences of categorical personality disorders, however, our research suggests that problems in social functioning might continue to predict further impairments in personality functioning across adolescence. Thus, managing the risk of personality pathology would not only include strengthening of the supportive network of the adolescent social world, but also continued efforts to reduce discordant relationships aspects with adults which prove to have a deteriorating effect on personality functioning independent of adolescent age.

## Supplementary Information

**Additional file 1.**

## Data Availability

The datasets used and/or analyzed during the current study are available from the corresponding author on reasonable request.

## References

[1] American Psychiatric Association. Diagnostic and Statistical Manual of Mental Disorders (DSM-5). 5th ed. Am. Psychiatr. Assoc. Washington, DC: American Psychiatric Publishing; 2013.

[2] Tyrer P, Mulder R, Kim Y-R, Crawford MJ (2019). The development of the ICD-11 classification of personality disorders: an amalgam of science, pragmatism, and politics. Annu Rev Clin Psychol.

[3] Morey LC, Bender DS, Skodol AE. Validating the proposed diagnostic and statistical manual of mental disorders, 5th edition, severity indicator for personality disorder. J Nerv Ment Dis. 2013;201:729–35. 10.1097/NMD.0b013e3182a20ea8. 10.1097/NMD.0b013e3182a20ea8 23995027

[4] World Health Organization (2018). International Statistical Classification of Diseases and Related Health Problems, 11th Revision (ICD-11).

[5] Weekers LC, Verhoeff SCE, Kamphuis JH, Hutsebaut J (2021). Assessing criterion a in adolescents using the semistructured interview for personality functioning DSM-5. Personal Disord United States.

[6] Sharp C, Vanwoerden S, Wall K. Adolescence as a sensitive period for the development of personality disorder. Psychiatr Clin North Am. Elsevier Inc; 2018;41:669–83. 10.1016/j.psc.2018.07.004. 10.1016/j.psc.2018.07.004 30447731

[7] Sharp C. Adolescent personality pathology and the alternative model for personality disorders: self development as Nexus. Psychopathology. 2020;1–7. 10.1159/000507588. 10.1159/000507588 32464626

[8] Erikson HE (1968). Identity: Youth and crisis.

[9] Pasupathi M, Hoyt T (2009). The development of narrative identity in late adolescence and emergent adulthood: the continued importance of listeners. Dev Psychol.

[10] Allen PJ, Loeb LE (2015). The autonomy-connection challange in adolescent-peer relationships. Child Dev Perspect.

[11] Pfeifer JH, Allen NB. Puberty Initiates Cascading Relationships Between Neurodevelopmental, Social, and Internalizing Processes Across Adolescence. Biol Psychiatry. Elsevier Inc; 2021;89:99–108. 10.1016/j.biopsych.2020.09.002. 10.1016/j.biopsych.2020.09.002 PMC8494463 33334434

[12] Cierpka A (2014). Narrative identity of adolescents and family functioning. Psychol Lang Commun.

[13] Vijayakumar N, Pfeifer JH (2020). Self-disclosure during adolescence: exploring the means, targets, and types of personal exchanges. Curr Opin Psychol Elsevier Ltd.

[14] Meeus W (2011). The study of adolescent identity formation 2000–2010: A review of longitudinal research. J Res Adolesc.

[15] de Moor EL, Van der Graaff J, van Doeselaar L, Klimstra TA, Branje S (2021). With a little help from my friends? Perceived friendship quality and narrative identity in adolescence. J Res Adolesc.

[16] Hill NE, Bromell L, Tyson DF, Flint R (2007). Developmental commentary: Ecological perspectives on parental influences during adolescence. J Clin Child Adolesc Psychol.

[17] Boele S, Van der Graaff J, de Wied M, Van der Valk IE, Crocetti E, Branje S. Linking Parent–Child and Peer Relationship Quality to Empathy in Adolescence: A Multilevel Meta-Analysis. J Youth Adolesc. Springer US; 2019;48:1033–55. 10.1007/s10964-019-00993-5. 10.1007/s10964-019-00993-5 PMC6525137 30810858

[18] Stern JA, Costello MA, Kansky J, Fowler C, Loeb EL, Allen JP (2021). Here for you: attachment and the growth of empathic support for friends in adolescence. Child Dev.

[19] Davis K. Friendship 2.0: Adolescents’ experiences of belonging and self-disclosure online. J Adolesc. Elsevier Ltd; 2012;35:1527–36. 10.1016/j.adolescence.2012.02.013. 10.1016/j.adolescence.2012.02.013 22475444

[20] Bauminger N, Finzi-Dottan R, Chason S, Har-Even D (2008). Intimacy in adolescent friendship: the roles of attachment, coherence, and self-disclosure. J Soc Pers Relat.

[21] Benson MJ, Mcwey LM, Ross JJ (2006). Parental attachment and peer relations in adolescence : a meta-analysis. Res Hum Dev.

[22] Schwartz OS, Simmons JG, Whittle S, Byrne ML, Yap MBH, Sheeber LB (2017). Affective parenting behaviors, adolescent depression, and brain development: a review of findings from the orygen adolescent development study. Child Dev Perspect.

[23] McDougall P, Vaillancourt T (2015). Long-term adult outcomes of peer victimization in childhood and adolescence: Pathways to adjustment and maladjustment. Am Psychol.

[24] Vanwoerden S, Leavitt J, Gallagher MW, Temple JR, Sharp C (2019). Dating violence victimization and borderline personality pathology: Temporal associations from late adolescence to early adulthood. Personal Disord.

[25] Platt B, Kadosh KC, Lau JYF (2013). The role of peer rejection in adolescent depression. Depress Anxiety.

[26] Carlson EA, Egeland B, Sroufe LA (2009). A prospective investigation of the development of borderline personality symptoms. Dev Psychopathol.

[27] Stepp SD, Lazarus SA, Byrd AL (2016). A systematic review of risk factors prospectively associated with borderline personality disorder: taking stock and moving forward. Personal Disord.

[28] Sharp C, Venta A, Vanwoerden S, Schramm A, Ha C, Newlin E, et al. First empirical evaluation of the link between attachment, social cognition and borderline features in adolescents. Compr Psychiatry. Elsevier Inc.; 2016;64:4–11. 10.1016/j.comppsych.2015.07.008. 10.1016/j.comppsych.2015.07.008 26298843

[29] Vanwoerden S, Kalpakci A, Sharp C (2017). The relations between inadequate parent-child boundaries and borderline personality disorder in adolescence. Psychiatry Res Elsevier Ireland Ltd.

[30] Hessels CJ, van den Berg T, Lucassen SA, Laceulle OM, van Aken MAG. Borderline personality disorder in young people: associations with support and negative interactions in relationships with mothers and a best friend. Borderline Personal Disord Emot Dysregulation. Borderline Personality Disorder and Emotion Dysregulation; 2022;9:1–11. 10.21203/rs.3.rs-868127/v1. 10.1186/s40479-021-00173-7 PMC8734252 34986894

[31] Haltigan JD, Vaillancourt T (2016). Identifying trajectories of borderline personality features in adolescence: antecedent and interactive risk factors. Can J Psychiatry.

[32] Hatkevich C, Mellick W, Reuter T, Temple JR, Sharp C (2017). Dating violence victimization, nonsuicidal self-injury, and the moderating effect of borderline personality disorder features in adolescent inpatients. J Interpers Violence.

[33] Reuter TR, Sharp C, Temple JR, Babcock JC (2015). The relation between borderline personality disorder features and teen dating violence. Psychol Violence.

[34] Barkauskienė R, Skabeikytė G, Gervinskaitė-Paulaitienė L. The Role of Borderline Personality Symptoms for Psychosocial and Health Related Functioning among Adolescents in a Community Sample. Child Youth Care Forum. Springer US; 2021;50:437–52. 10.1007/s10566-020-09581-2.

[35] Winograd G, Cohen P, Chen H. Adolescent borderline symptoms in the community: prognosis for functioning over 20 years. J Child Psychol Psychiatry. England; 2008;49:933– 10.1111/j.1469-7610.2008.01930.x 18665882

[36] Vanwoerden S, Franssens R, Sharp C, De Clercq B. The Development of Criterion A Personality Pathology: The Relevance of Childhood Social Functioning for Young Adult Daily Self-Functioning. Child Psychiatry Hum Dev. Springer US; 2021; 10.1007/s10578-021-01187-6. 10.1007/s10578-021-01187-6 PMC8859861 34076800

[37] Furman W, Buhrmester D (1985). Children’s perceptions of the personal relationships in their social networks. Dev Psychol.

[38] McLean KC, Jennings LE (2012). Teens telling tales: How maternal and peer audiences support narrative identity development. J Adolesc Elsevier Ltd.

[39] Sapiro B, Ward A (2020). Marginalized youth, mental health, and connection with others: a review of the literature. Child Adolesc Soc Work J.

[40] Choate AM, Fatimah H, Bornovalova MA (2021). Comorbidity in borderline personality: understanding dynamics in development. Curr Opin Psychol Elsevier Ltd.

[41] Widiger TA, Samuel DB (2005). Diagnostic categories or dimensions? a question for the diagnostic and statistical manual of mental disorders–fifth edition. J Abnorm Psychol United States.

[42] Goth K, Birkhölzer M, Schmeck K (2018). Assessment of personality functioning in adolescents with the LoPF–Q 12–18 self-report questionnaire. J Pers Assess Routledge.

[43] Barkauskiene R, Skabeikyte G. Culture-adapted Lithuanian version of the self-report questionnaire LoPF-Q 12–18 (Levels of Personality Functioning Questionnaire; authors Goth & Schmeck) - short manual. Offenbach: academic-tests; 2020.

[44] Cosgun S, Goth K, Cakiroglu S. Levels of Personality Functioning Questionnaire (LoPF-Q) 12–18 Turkish Version: Reliability, Validity, Factor Structure and Relationship with Comorbid Psychopathology in a Turkish Adolescent Sample. J Psychopathol Behav Assess. 2021;43:620–31. 10.1007/s10862-021-09867-2.

[45] Kerr S, Mclaren V, Cano K, Vanwoerden S, Goth K, Sharp C. Levels of personality functioning questionnaire 12–18 (LoPF-Q 12–18): factor structure, validity, and clinical cut-offs. Assessment. 2022;0:1–13. 10.1177/10731911221124340. 10.1177/10731911221124340 PMC10200067 36124366

[46] Furman W. The measurement of friendship perceptions: Conceptual and methodological issues. Co they keep Friendsh Child Adolesc. 1998;41–65.

[47] Achenbach TM, Rescorla LA. Manual for the ASEBA school-age forms & profiles: an intengrated system of multi-informant assessment. Burlington; 2001.

[48] Žukauskienė R, Kajokienė I, Vaitkevičius R. Mokyklinio amžiaus vaikų ASEBA klausimynų (CBCL6/18, TRF6/18, YSR11/18) vadovas. Vilnius; 2012.

[49] Bach B, First MB (2018). Application of the ICD-11 classification of personality disorders. BMC Psychiatry.

[50] De Clercq B, Van Leeuwen K, Van Den Noortgate W, De Bolle M, De Fruyt F (2009). Childhood personality pathology: Dimensional stability and change. Dev Psychopathol.

[51] Sharp C, Vanwoerden S, Schmeck K, Birkhölzer M, Goth K. An Evaluation of Age-Group Latent Mean Differences in Maladaptive Identity in Adolescence. Front Psychiatry. 2021;12. 10.3389/fpsyt.2021.730415. 10.3389/fpsyt.2021.730415 PMC8484521 34603108

[52] Crick N, Murray-Close D, Woods K (2005). Borderline personality features in childhood: a short-term longitudinal study. Dev Psychopathol.

[53] Skabeikyte G, Barkauskiene R. A systematic review of the factors associated with the course of borderline personality disorder symptoms in adolescence. Borderline Personal Disord Emot Dysregulation. 2021;8:1–11. 10.1186/s40479-021-00151-z. 10.1186/s40479-021-00151-z PMC8054370 33866976

[54] Dixon-Gordon KL, Whalen DJ, Scott LN, Cummins ND, Stepp SD. The Main and Interactive Effects of Maternal Interpersonal Emotion Regulation and Negative Affect on Adolescent Girls’ Borderline Personality Disorder Symptoms. Cognit Ther Res. Springer US; 2016;40:381–93. 10.1007/s10608-015-9706-4. 10.1007/s10608-015-9706-4 PMC4866813 27185969

[55] Stepp SD, Whalen DJ, Scott LN, Zalewski M, Loeber R, Hipwell AE (2014). Reciprocal effects of parenting and borderline personality disorder symptoms in adolescent girls. Dev Psychopathol United States.

[56] Munson MR, Brown S, Spencer R, Edguer M, Tracy E (2015). Supportive relationships among former system youth with mental health challenges. J Adolesc Res.

[57] Luijten CC, van de Bongardt D, Jongerling J, Nieboer AP (2021). Associations between adolescents’ internalizing problems and well-being: is there a buffering role of boys’ and girls’ relationships with their mothers and fathers?. BMC Public Health.

[58] Villalobos Solís M, Smetana JG, Comer J (2015). Associations among solicitation, relationship quality, and adolescents’ disclosure and secrecy with mothers and best friends. J Adolesc Elsevier Ltd.

[59] van Harmelen A-L, Kievit RA, Ioannidis K, Neufeld S, Jones PB, Bullmore E (2017). Adolescent friendships predict later resilient functioning across psychosocial domains in a healthy community cohort. Psychol Med.

[60] Kendrick K, Jutengren G, Stattin H (2012). The protective role of supportive friends against bullying perpetration and victimization. J Adolesc Elsevier Ltd.

[61] Lazarus SA, Choukas-Bradley S, Beeney JE, Byrd AL, Vine V, Stepp SD. Too Much Too Soon?: Borderline Personality Disorder Symptoms and Romantic Relationships in Adolescent Girls. J Abnorm Child Psychol. 2019;47:1995–2005. 10.1007/s10802-019-00570-1. 10.1007/s10802-019-00570-1 PMC7045362 31240430

[62] Akiyama H, Antonucci T, Takahashi K, Langfahl ES. Negative interactions in close relationships across the life span. Journals Gerontol. Ser. B Psychol.; 2003.70–9. 10.1093/geronb/58.2.P70. 10.1093/geronb/58.2.p70 12646590

[63] Chanen A, Sharp C, Hoffman P (2017). Prevention and early intervention for borderline personality disorder: a novel public health priority. World Psychiatry.

