# Supplementary material for: Personality pathology in adolescence: relationship quality with parents and peers as predictors of the level of personality functioning
Source: Borderline Personal Disord Emot Dysregul. 2022 Oct 19;9:31. doi: 10.1186/s40479-022-00202-z (PMC9579636; doi:10.1186/s40479-022-00202-z)
Supplement: Supplementary file 1 — Additional file 1. [file 40479_2022_202_MOESM1_ESM.docx]

**Personality pathology in adolescence: relationship quality with parents and peers as predictors of the level of personality functioning**

Gabriele Skabeikyte-Norkiene^*,1^, Carla Sharp^2^, Paulina Anna Kulesz^3^ and Rasa Barkauskiene^4^

^1^Institute of Psychology, Faculty of Philosophy, Vilnius University, Lithuania; [gabriele.skabeikyte@fsf.vu.lt](mailto:gabriele.skabeikyte@fsf.vu.lt); ORCID iD <https://orcid.org/0000-0002-6404-0445>.

^2^ Department of Psychology, University of Houston; [csharp2@Central.UH.EDU](mailto:csharp2@Central.UH.EDU); ORCID iD <https://orcid.org/0000-0001-8349-4701>

^3^ Texas Institute for Measurement, Evaluation, and Statistics (TIMES), University of Houston; [Paulina.Kulesz@times.uh.edu](mailto:Paulina.Kulesz@times.uh.edu); ORCID iD <https://orcid.org/0000-0002-6372-794X>

^4^Institute of Psychology, Faculty of Philosophy, Vilnius University, Lithuania; [rasa.barkauskiene@fsf.vu.lt](mailto:rasa.barkauskiene@fsf.vu.lt); ORCID iD <https://orcid.org/0000-0002-4464-2481>.

*Address correspondence to: Gabriele Skabeikyte-Norkiene, Institute of Psychology, Faculty of Philosophy, Vilnius University, Universiteto st. 9, LT-01513, Vilnius, Lithuania. E-mail address: [gabriele.skabeikyte@fsf.vu.lt](mailto:gabriele.skabeikyte@fsf.vu.lt).

**Abstract:** *Background.* The dimensional approach to personality pathology opens up the possibility to investigate adolescence as a significant period for the development of personality pathology. Recent evidence suggests that symptoms of personality pathology may change during adolescence, but the negative consequences such as impaired social functioning persist later on in life. Thus, we think that problems in social functioning may further predict personality impairments. The current study aimed at investigating the role of relationship quality with parents and peers for the prediction of the level of personality functioning across adolescence. We hypothesized that 1) relationship quality with both parents and peers will significantly account for the level of personality functioning in adolescence and 2) the importance of relationship quality with peers for the relation to impairments in personality functioning will increase with age. *Methods.* A community sample consisting of 855 adolescents aged 11-18 (M = 14.44, SD = 1.60; 62.5% female) from different regions in Lithuania participated in this study. Self-report questionnaires included the *Levels of Personality Functioning Questionnaire* to investigate personality impairments and the *Network of Relationships Questionnaire* to assess the quality of dyadic relationships. *Results.* Discord in the parent, but not peer relationships, was related to a more severe level of personality functioning across adolescence. Lower levels of closeness with parents accounted for higher impairments in personality functioning. The importance of closeness with peers for the explanation of the level of personality functioning increased with age. *Conclusions.* During the sensitive period for the development of a personality disorder, relationship quality with the closest adults and peers both remain important for the explanation of impairments in personality functioning. *Trial registration:* Not applicable.

Keywords: Level of personality functioning, Alternative Model for Personality Disorders (AMPD), ICD-11, Adolescence, Relationship quality, Network of relationships

**Declarations**

**Ethics approval and consent to participate:** The studies involving human participants were reviewed and approved by Vilnius University Psychological Research Ethics Committee. Written informed consent to participate in this study was provided by the participant‘s legal guardian/next of kin.

**Consent for publication:** Not applicable.

**Availability of data and material:** The datasets used and/or analyzed during the current study are available from the corresponding author on reasonable request.

**Competing interests:** The authors declare that they have no competing interests.

**Funding:** This study was funded by a grant (No. S-MIP-21-20) from the Research Council of Lithuania.

**Authors’contributions:** GS-N: conceptualization, data collection, data analysis, and writing the initial draft. CS: contribution to the introduction, results, and discussion part of the paper. PK: data analysis, contribution to the results section of the paper, preparation of figures. RB: conceptualization, contribution to all parts of the paper, reviewing, and writing. All authors contributed to the article and approved the submitted version.
